# Discriminating Non-native Vowels on the Basis of Multimodal, Auditory or Visual Information: Effects on Infants’ Looking Patterns and Discrimination

**DOI:** 10.3389/fpsyg.2016.00525

**Published:** 2016-04-19

**Authors:** Sophie Ter Schure, Caroline Junge, Paul Boersma

**Affiliations:** ^1^Linguistics, University of AmsterdamAmsterdam, Netherlands; ^2^Experimental Psychology, Utrecht UniversityUtrecht, Netherlands

**Keywords:** audiovisual speech integration, distributional learning, multimodal perception, infants, non-native phonemes, gaze locations, intersensory redundancy hypothesis, language acquisition

## Abstract

Infants’ perception of speech sound contrasts is modulated by their language environment, for example by the statistical distributions of the speech sounds they hear. Infants learn to discriminate speech sounds better when their input contains a two-peaked frequency distribution of those speech sounds than when their input contains a one-peaked frequency distribution. Effects of frequency distributions on phonetic learning have been tested almost exclusively for *auditory* input. But auditory speech is usually accompanied by *visual* information, that is, by visible articulations. This study tested whether infants’ phonological perception is shaped by distributions of visual speech as well as by distributions of auditory speech, by comparing learning from multimodal (i.e., auditory–visual), visual-only, or auditory-only information. Dutch 8-month-old infants were exposed to either a one-peaked or two-peaked distribution from a continuum of vowels that formed a contrast in English, but not in Dutch. We used eye tracking to measure effects of distribution and sensory modality on infants’ discrimination of the contrast. Although there were no overall effects of distribution or modality, separate *t*-tests in each of the six training conditions demonstrated significant discrimination of the vowel contrast in the two-peaked multimodal condition. For the modalities where the mouth was visible (visual-only and multimodal) we further examined infant looking patterns for the dynamic speaker’s face. Infants in the two-peaked multimodal condition looked longer at her mouth than infants in any of the three other conditions. We propose that by 8 months, infants’ native vowel categories are established insofar that learning a novel contrast is supported by attention to additional information, such as visual articulations.

## Introduction

Infants’ perception of speech sound contrasts is modulated by their language environment. Their perception of contrasts that are non-native to their mother tongue declines in the second half of the 1st year, while their perception of native contrasts remains or improves (e.g., Kuhl et al., 2006). This process of perceptual narrowing is influenced by various characteristics of the speech input: for instance, the frequency of the speech sounds, their acoustic salience and their statistical distributions. A decline in the perception of non-native contrasts happens faster for sounds that occur more frequently in a particular language (Anderson et al., 2003), and some salient non-native contrasts remain discriminable after the 1st year (Best et al., 1988) while some non-salient native contrasts require more than 6 months of exposure to become discriminable (e.g., Narayan et al., 2010). Also, perceptual narrowing might occur earlier for vowels than for consonants (e.g., Polka and Werker, 1994, although to our knowledge the literature has not yet reported any direct statistical comparisons between the two phoneme classes). Although the frequency, saliency and major class (vowel or consonant) of the speech sounds may be factors in perceptual narrowing, most language acquisition theories that aim to explain how infants acquire their native speech sounds focus on the mechanism of distributional learning (e.g., Pierrehumbert, 2003; Werker and Curtin, 2005; Kuhl et al., 2008). According to the distributional learning hypothesis, infants learn to discriminate a contrast on a particular continuum of auditory values better if the values that the child hears from this continuum follow a two-peaked frequency distribution than if these values follow a one-peaked distribution (e.g., Maye et al., 2008).

However, the input that infants receive contains more than just auditory information: language occurs in a rich sensory environment that also contains *visual* input. Some theories propose that visual cues congruent with speech sounds, like objects present when the speech sounds are uttered, or the mouth movements from the interlocutor, may help learning phonological categories by simply increasing infants’ attention to auditory contrasts (e.g., Kuhl et al., 2008). Yet, there is accumulating evidence that infants’ early phonological representations consist of both auditory *and* visual information. For example, 2-month-old infants notice a mismatch between speech sounds and a speaking face (Bristow et al., 2009) and infants between 2 and 5 months are able to match auditory and visual speech cues (Kuhl and Meltzoff, 1982; Patterson and Werker, 2003; Kushnerenko et al., 2008; Bristow et al., 2009). The type of audiovisual speech can also affect 6-month-olds’ listening preferences for tokens from a novel phonetic contrast: when speech sounds match with the visual information, infants prefer alternating tokens over repeated tokens, whereas when the speech sounds are incongruent with the visual information (i.e., point to different phonemes), they prefer repeated tokens over alternating tokens (Danielson et al., 2015). Pons et al. (2009) suggested that intersensory perception for non-native contrasts declines between 6 and 11 months: Spanish 6-month-olds are better than 11-month-olds at matching the non-native (English) [ba]∼[va] contrast to the corresponding visual articulations. Perceptual narrowing can even take place with visual speech in the absence of auditory information (Weikum et al., 2007): monolingual infants visually discriminate between their own language and another one better at 4 (and perhaps 6) months than at 8 months. Furthermore, infants are sensitive to the McGurk effect: when hearing a syllable [ba] while seeing someone pronounce [ga], 4.5- to 5-month-old infants, like adults, appear to perceive a fused percept /da/ instead of one of the played syllables (Rosenblum et al., 1997; Burnham and Dodd, 2004). This indicates that infants activate multimodal combinations of phonological features in perception. Together, these results suggest that phonological categories relate to visual cues as well as to auditory cues. This raises the question whether the co-presence of visual articulation information *improves* learning of a phonological contrast. Might it even be the case that infants’ emerging phonological categories can be affected by statistical distributions of visual articulations alone besides the statistical distributions of speech sounds (e.g., Maye et al., 2008)? This study aims to investigate in detail how (the added) visual articulation information influences distributional learning of a non-native vowel contrast.

So far, only one study tested distributional learning from auditory distributions in tandem with visual articulations (Teinonen et al., 2008). In that study, 6-month-old infants were exposed to a continuum of sounds from a phonological contrast that was familiar to them (/ba/∼/da/), but sounds from the middle of the continuum occurred more frequently. Infants who are familiarized with such a one-peaked frequency distribution of sounds typically discriminate between those sounds less well than infants who are familiarized with a two-peaked distribution (e.g., Maye et al., 2008). In the study of Teinonen et al. (2008), the speech sounds were accompanied by videotaped articulations. Half of the infants (one-category group) were presented with a video of just one visual articulation ([ba] or [da]) together with the one-peaked continuum, while the other half of the infants (two-category group) saw two visual articulations; one video of [ba] for sounds on the left side of the continuum, one video of [da] for sounds on the right side of the continuum. Infants in the two-category group subsequently discriminated the speech sounds somewhat better than infants in the one-category group. Apparently, the presence of two visual articulations can aid infants’ perception of a (native) phonological contrast. It seems plausible, then, that infants could also learn a *non-native* phonological contrast from audiovisual combinations, as long as the visual stream contains two visible articulations. Further, if infants are sensitive to distributions of auditory speech information, they may also be sensitive to the distributions of visual speech information. Hence, it would be revealing to compare learning from a two-peaked multimodal (e.g., visual with auditory) distribution with learning from a one-peaked multimodal distribution. To fully evaluate this distributional effect in a multimodal context, we compare this distributional effect also in the two unimodal sensory contexts: a visual-only and auditory-only learning context.

There is reason to belief that infants learn better in a multimodal context than in a unimodal context. According to the intersensory redundancy hypothesis (e.g., Bahrick and Lickliter, 2012), the combination of auditory and visual information originating from the same stimulus helps infants to attend to relevant events in their environment. This, in turn, facilitates learning from these events. From this hypothesis, we expect that infants would learn to discriminate a phonological contrast better from audiovisual information than from unimodal stimulation alone. Indeed, presentation with redundant multimodal speech cues facilitates auditory processing both in infants and adults (e.g., Hyde et al., 2010). Crucially, it is around the same time as when perceptual narrowing begins, that there is a change in infants’ looking behavior when scanning faces. From attending most to the eyes of a speaking face in the first 6 months, infants start to look more at the mouth area by 6–8 months (Hunnius and Geuze, 2004; Lewkowicz and Hansen-Tift, 2012). Lewkowicz and Hansen-Tift show that this mouth preference continues until at least 10 months of age for native speech and until at least 12 months for non-native speech (also Danielson et al., 2014), whereas adults again look more at the eyes.

Taking together findings on the effect of multimodal speech on infants’ gaze locations and learning, and the influence of frequency distributions on infants’ changing perception of speech sounds, the question arises whether infants’ learning of a novel speech contrast is facilitated in the presence of multimodal – auditory plus visual – distributions of speech sounds. To address this question, the current eyetracking study exposed 8-month-olds to a non-native vowel contrast in a typical distributional learning experiment: some infants were presented with a one-peaked distribution of the speech sounds, while others were presented with a two-peaked distribution of the same sounds. To find out whether visual distributions of speech influenced discrimination of the contrast, infants were further divided into one of three modality conditions: the vowel information was presented either only auditory, only visually, or through both modalities. Thus, there were six different familiarization conditions in total. After the familiarization phase, all infants followed a similar habituation and test paradigm (Maye et al., 2008) to assess whether they could discriminate the novel contrast (still presented only as auditory, as visual or as multimodal information).

Distributional learning for speech sounds has, so far, mostly been tested with consonant contrasts (e.g., Maye et al., 2002, 2008; Yoshida et al., 2010; Cristia et al., 2011). Because it emerges from the literature that infants attune to their native vowels slightly earlier than to their native consonants (Kuhl et al., 1992; Polka and Werker, 1994; Bosch and Sebastián-Gallés, 2003; for an overview see Tsuji and Cristia, 2014), it is possible that by 8 months their sensitivity to a non-native vowel contrast is not as susceptible to frequency distributions as it would be in the case of a consonant contrast (e.g., Yoshida et al., 2010). Indeed, the few auditory-only studies on distributional learning of vowels have yielded mixed results (a null result for distributional learning at 8 months, Pons et al., 2006b; a null result for distributional learning at 6 months, Pons et al., 2006a; an effect of distributional learning at 2 months, Wanrooij et al., 2014). Thus, by presenting infants with a non-native *vowel* contrast, we aim to create a situation in which any effects of distribution and modality of available cues surface in our testing paradigm. In other words, with a non-native vowel contrast we can assess whether multimodal speech information can improve learning in this difficult situation as compared to auditory-only speech information (e.g., Hyde et al., 2010; Bahrick and Lickliter, 2012). The non-native contrast we focus on is the English [ε] - [æ] contrast. Adult speakers of Dutch have difficulty perceiving the difference between the two English vowels, instead hearing only a vowel resembling Dutch /ε/. Even in an English word recognition context, Dutch adults initially activate only words with /ε/ for items that contain either [ε] or [æ] (Weber and Cutler, 2004). Thus, this English vowel contrast is a perfect test case for distributional learning with Dutch infants (Wanrooij et al., 2014; Ter Schure et al., in press).

Given that previous studies on novel vowel learning in infants aged 6 months or older failed to show any effect of auditory frequency distributions, we reasoned that at 8 months, successful distributional learning of vowels requires more than just 2 min of auditory exposure, and that additional, visual speech information would support the learning process. We therefore predict infants exposed to a two-peaked multimodal distribution to show better learning than infants exposed to a one-peaked multimodal distribution, and better learning than infants exposed to a two-peaked auditory distribution. With regard to our expectations for infants in the visual condition, these are less clear: our study is the first to test learning of a phonological contrast from silent articulations. There is evidence that infants are sensitive to visual distributions of objects (Raijmakers et al., 2014), and that perceptual narrowing occurs for silent visual speech (Weikum et al., 2007). However, none of these studies look at learning phonological contrasts. To create the best opportunity to learn a non-native contrast from the visual articulations, we presented infants in our visual condition with the same synchronous audiovisual stimuli as we presented to infants in the multimodal condition. In this way, infants’ attention during the test should remain equal across conditions (e.g., Ter Schure et al., 2014). However, for the visual group, the speech signal was stripped of all contrastive formant information. Only the intensity and pitch contours remained, which ensured a synchronous on- and offset with the opening and closing of the speaking mouth. In this way, we hoped that infants in the two-peaked visual-only condition would be able to learn the phonological contrast as well as infants in the two-peaked auditory-only and multimodal groups. Similarly, to ensure the highest possible level of attention from the infants in the auditory condition, they saw the same dynamic face as infants in the visual and multimodal conditions, but the mouth was covered by the hand of the speaker.

We further reasoned that if infants are sensitive to the visual articulatory cues, this should be reflected in their gaze patterns in the training phase. We expect infants in the multimodal conditions to attend more to the mouth than infants in the other two conditions, if redundancy between the senses guides infants’ attention when presented with a speaking face (e.g., Bahrick and Lickliter, 2012). Further, on the basis of recent findings on infants’ gaze location when presented with a speaking face (Lewkowicz and Hansen-Tift, 2012; Tomalski et al., 2013; Danielson et al., 2014), we expect that infants in the two-peaked conditions look more at the mouth than infants in the one-peaked conditions; for them, the speech stimuli would form a new phonological contrast, while for infants in the one-peaked condition, the speech stimuli would correspond to their native language input. It is possible that we only observe effects of distribution on infants’ fixations at a later stage in the training phase, because it requires time for the type of distribution to become apparent. We therefore divided the training phase into two blocks to evaluate whether infants’ fixations to the speaker’s mouth and eyes changed as a function of time.

To sum up, our hypothesis is that multimodal speech information provides a better opportunity to learn a non-native phonological contrast than auditory-only or visual-only information, because the synchrony between articulations and speech sounds increase infants’ attention to the contrast (e.g., Bahrick and Lickliter, 2012). According to the distributional learning hypothesis (e.g., Maye et al., 2008), infants presented with a two-peaked training distribution should discriminate the vowel contrast better at test than infants presented with a one-peaked training distribution. If visual speech cues *improve* phonological learning, we expect better learning in the two-peaked multimodal condition than in the two-peaked auditory-only condition. If visual speech cues are *sufficient* for learning a phonological contrast, we expect better learning in the two-peaked visual condition than in the one-peaked visual condition. Our hypothesis for infants’ gaze behavior when learning a non-native contrast is that multimodal speech information increases infants’ attention to the mouth area as compared to visual-only speech information, and that a two-peaked training distribution increases attention to the mouth as compared to a one-peaked training distribution.

## Materials and Methods

### Participants

A total of 167 monolingual Dutch-hearing infants aged between 7.5 and 8.5 months were tested in this study. Only infants who provided data for the full course of the experiment were included in the analysis (*N* = 93). Infants were randomly assigned to a *multimodal*, a *visual*, and an *auditory* training condition. The final groups consisted of 36 infants in the multimodal condition (mean age = 8;01 months, range 7;14–8;14 months, 15 girls), 29 infants in the visual condition (mean age = 8;0 months, range 7;11–8;15 months, 16 girls) and 28 infants in the auditory condition (mean age = 7;29 months, range 7;17–8;21 months, 13 girls). All infants were exposed to sounds and/or visual articulations from the same phonetic continuum, but within each modality condition, this phonetic continuum was either one-peaked or two-peaked; thus, there were six different groups in total. In the multimodal condition, 18 infants were presented with a one-peaked continuum and 18 infants were presented with a two-peaked continuum. In the visual condition, there were 14 infants in the one-peaked group and 15 infants in the two-peaked group. In the auditory condition, there were 15 infants in the one-peaked group and 13 infants in the two-peaked group.

Ethical permission to conduct the study was given by the ethical committee of the psychology department of the University of Amsterdam. All parents provided written informed consent. Infants came from Dutch-speaking families, were born full term (37–42 weeks) and had no history of language- or hearing problems. Another 74 infants were tested but excluded from the analysis because of equipment failure (*n*_vis_ = 3, *n*_aud_ = 13), not attending to at least 50% of the training trials (*n*_multi_ = 15, *n*_vis_ = 11, *n*_aud_ = 18), or not meeting the habituation criterion (*n*_multi_ = 11, *n*_vis_ = 3). Note that more infants from the multimodal condition were excluded for staying focused during the whole habituation phase and therefore failing to meet the habituation criterion than infants from the other conditions: in the multimodal condition, this was 11 infants out of a total number of 62 tested infants (*n*_1-peak_ = 2, *n*_2-peak_ = 9); in the visual condition, 3 out of 46 tested infants (*n*_1-peak_ = 1, *n*_2-peak_ = 2), and in the auditory condition, 0 out of 59 tested infants (difference between conditions *p* = 0.001, three-by-two Fisher’s exact test).

### Stimuli

Visual and auditory instances of a female speaker saying /fεp/ and /fæp/ were manipulated to create an audiovisual continuum of 32 steps: from a clear token of /ε/ via ambiguous sounds to a clear token of /æ/. Vowels were embedded in a /f_p/-consonant context. Syllables were 830 ms long, with the vowel 266 ms.

The auditory vowel continuum was created with the Klatt synthesizer in the Praat software (Boersma and Weenink, 2011). Endpoints for the continuum were based on average values of Southern British /æ/ and /ε/ reported in Deterding (1997) and chosen so that the /æ/-sound did not overlap with average *F*1-values for Dutch /a/ (Adank et al., 2004): the minimum *F*1-value was 12.5 ERB^1^ (689 Hz) and the maximum *F*1-value was 15.5 ERB (1028 Hz). F2 ranged from 20.2 to 20.8 ERB; stimuli with lower *F*1 values had higher *F*2 values and vice versa.

**FIGURE 1:**
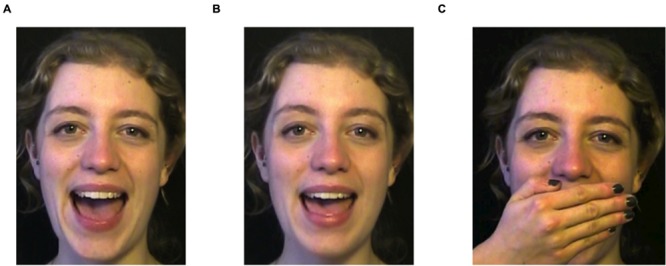
**Stills from the training videos. (A)** and **(B)** are taken from video 1 and video 32 in the multimodal and visual conditions. **(C)** is taken from video 11 from the auditory condition, in which infants saw no visual articulation information.

To create the visual vowel continuum, a female speaker of Southern British English was recorded while she repeated the syllables /fæp/ and /fεp/ in infant-directed speech. Facial expressions (distance between nose and eyebrows, mouth opening, lip width) were measured in pixels and instances of /fæp/ and /fεp/ were paired to find the best matching set of two videos. From those two videos, the vowel portion was spliced and exported as individual picture frames. These frames were imported two-by-two – first frame of [æ] with first frame of [ε], and so on – into the morphing software MorphX (Wennerberg, 2011). With linear interpolation a 30-step continuum was made between each set of frames, resulting in 32 videos: step 1 a clear instance of /æ/, step 2 slightly closer to /ε/, steps 16 and 17 ambiguous instances, and step 32 a clear instance of /ε/ (see **Figure 1**). A third video provided the /f_p/-context for the vowels. In a pilot experiment, it was established that native British English speakers (*n* = 11) could identify the two endpoint vowels in a categorization task on the basis of only visual articulatory information (mean proportion correct 0.65, range 0.54–0.75, significantly different from chance at 0.50 with *SD* = 0.07).

Infants in the visual condition heard the same syllables as infants in the multimodal and auditory conditions, but with all formant information except the intonation contour removed. Pink noise was added for the full duration of the experiment to make the lack of vowel information appear more natural. Infants in the auditory condition saw the same videos as infants in the multimodal and visual conditions, but with a hand placed before the mouth of the speaking woman (**Figure 1**, picture C), so that the articulatory information was no longer visible.

**FIGURE 2:**
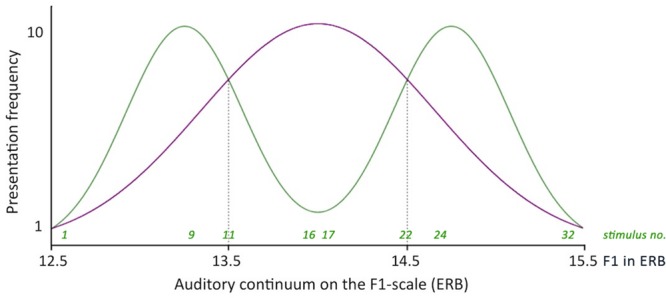
**Simplified frequency distributions of the one- and the two-peaked 32-step auditory continuum.** Dotted lines indicate the intersections between the two distributions, which correspond to stimuli 11 and 22 on the continuum (the test stimuli).

The frequency distributions of the 32-step continuum were manipulated to ensure that infants in the one-peaked group were exposed to a distribution approaching a one-peaked Gaussian curve with a mean of 14 ERB and a standard deviation of 0.66 ERB (see **Figure 2**). Infants in the two-peaked group were exposed to a distribution approaching a two-peaked Gaussian curve with local means of 13.25 and 14.75 ERB and a standard deviation of 0.33 ERB. The frequency curves of the one-peaked and two-peaked distributions met at 13.5 and 14.5 ERB. Stimuli with these values were presented to infants in both distribution groups with equal frequency (five times each).

### Apparatus

Infants were placed in a car seat in a soundproofed booth with their parent sitting behind them. Parents were instructed not to interact with their child during the trials. Stimuli were shown on the 17-inch monitor of the eye tracker, positioned 65 cm away from the infant’s face. Stimulus presentation and data collection were controlled by E-prime (Psychology Software Tools, Sharpsburg, PA, USA). A Tobii-120 Eye Tracker, sampling at 60 Hz, measured the infant’s eye gaze after a 5-point calibration of the participants’ eye characteristics. Sound was played through two speakers located on both sides of the monitor at a level of 65 dB.

### Procedure

#### Training

In the training phase, all infants were exposed to the 32 audiovisual stimuli. Each stimulus was shown between 1 and 10 times depending on the distribution group. In total, infants saw 128 stimuli during the training phase, presented in random order. Both test stimuli occurred exactly five times during training. A brief attention getter (consisting of a twirling star, a popping snowflake, or a shaking duck) was played if the infant looked away from the screen for 1.5 s or more. The experimenter terminated the attention getter (by starting the next trial) as soon as the infant looked back to the screen. All infants were presented with audiovisual stimuli; for infants in the auditory condition only the visual vowel information was obscured (panel C in **Figure 1**), while for infants in the visual condition, only the auditory vowel information was obscured (**Figures 1A,B**).

#### Habituation

After the training phase, we assessed discrimination of the vowel contrast using a habituation paradigm with a moving window of three trials and a maximum number of 25 trials. One full habituation trial consisted of eight repetitions of one stimulus from the training set (either stimulus 11 or 22). Habituation was completed when looking time on three subsequent trials fell below 50% compared to looking time during the first three habituation trials. As during training, the habituation stimuli contained auditory, visual, or multimodal vowel information, dependent on modality condition. The trial was terminated when the infant looked away for 2 s. An attention getter was played before the next trial started.

#### Test

Testing began immediately after the infant reached the habituation criterion. The test phase consisted of two ‘switch’ and two ‘same’ trials. As during habituation, each full test trial consisted of eight repetitions of the same stimulus; the trial was terminated when no look was recorded for 2 s, and followed by the attention getter. If stimulus 11 was used as the habituation stimulus, the ‘switch’ trial contained repetitions of stimulus 22 and the ‘same’ trial contained repetitions of stimulus 11. If stimulus 22 was the habituation stimulus, the ‘switch’ trial contained stimulus 11 and the ‘same’ trial stimulus 22. The order of the test trials was interleaved and counterbalanced between groups. Longer looks at ‘switch’ than at ‘same’ trials are interpreted as evidence of infants’ sensitivity to the contrast between the vowels. Note that for the visual modality conditions, the sound comprised the same (non-informative) token with pink noise throughout the experiment, which was paired with different tokens of the visual articulations from the /fæp/-/fεp/ continuum.

### Analysis

The data was cleaned for eye blinks prior to analysis. The average duration of infant eye blinks is 419 ms (Bacher and Smotherman, 2004) but we used a conservative time window of 250 ms (Olsen, 2012) to interpolate missing data. Gaps of missing data longer than 250 ms were coded as missing.

To measure differences in attention during training, we calculated the number of training trials that each infant looked at for 500 ms or more. Also, we calculated the number of habituation trials required to reach the habituation criterion. All measures were entered separately into a two-way analysis of variance (ANOVA) with modality condition (multimodal, visual, or auditory) and distribution group (one-peaked or two-peaked) as between-subjects factors.

Next, results from the test phase allowed us to assess whether infants can learn a vowel contrast from multimodal vs. unimodal frequency distributions. As dependent variables we calculated looking time differences between each pair of ‘same’ and ‘switch’ trials during the test phase (switch minus same; two pairs in total). These difference scores were entered into a repeated-measures ANOVA with test block as a within-subjects factor, and Modality (multimodal, visual, or auditory condition) and Distribution (one-peaked or two-peaked group) as between-subjects factors.

Finally, we explored whether infants’ visual scanning during the course of the training phase depended on the type of training they received. There were two regions of interest (ROI): the mouth and the eyes. Recall that in both the multimodal and the visual groups, the sound and the mouth movement were synchronous while for the auditory conditions the mouth movements were obscured by the speaker’s hand. We therefore only compared scanning patterns for those conditions in which both the mouth and eyes were visible (i.e., excluding the auditory conditions, because there the mouth was absent^2^). We calculated total looking time to mouth and to eyes as a proportion of total looking time to the face, in the first vs. second block of the training phase (64 trials per block). For both ROIs we entered these proportions in a repeated-measures ANOVA with training block (1 or 2) as a within-subject variable, and Modality condition (multimodal or visual) and Distribution group (one-peaked or two-peaked) as between-subjects factors.

## Results

### Attentional Differences during Training and Habituation

For the training phase, the dependent variable was the number of trials that infants attended to during the training phase (see **Table 1** for an overview). Infants did not differ on this measure [no interaction of Modality and Distribution, *F*(2,87) = 2.049, *p* = 0.135, nor any main effects]. On average, infants attended to 89.7 out of a maximum of 128 training trials (*SD* = 17.2). For the habituation phase, the dependent variable was the number of trials required to reach the habituation criterion (a 50% decline in looking time; cf. **Table 1**). Again, we did not observe any significant differences between groups [no interaction of Modality and Distribution, *F*(2,87) = 1.530, *p* = 0.222, nor any main effects]. On average, infants habituated within 13 trials (*SD* = 6.9).

**Table 1:** Attentional measures for each condition: the average number of trials that infants attended to for at least 500 ms during training, and the average number of trials required to reach habituation.

Modality	Distribution	*N*	Training trials *M*	*SD*	Habituation trials *M*	*SD*
Multimodal	1-peaked	18	88.3	17.9	12.3	5.1
	2-peaked	18	85.0	12.5	10.4	5.7
Visual	1-peaked	14	97.8	17.4	13.4	6.9
	2-peaked	15	85.2	10.8	13.6	7.6
Auditory	1-peaked	15	89.1	23.7	12.8	8.2
	2-peaked	13	94.6	16.8	16.8	8.2

Together, these two measures did not indicate that differences in the test phase were caused by general attentional differences between groups.

### Discrimination of the Vowel Contrast at Test

To measure discrimination of the vowel contrast at test, we calculated difference scores for two testing blocks, composed of looking times at ‘switch’ trials minus looking times at ‘same’ trials. If these scores are significantly different from zero, we can conclude that infants perceive a difference between the two vowel categories that were presented in these trials.

A 3-by-2 repeated-measures ANOVA with Modality (multimodal; visual; auditory) and Distribution (one-peaked; two-peaked) as between-subjects factors, and test block (2) as within-subjects factor, yielded no significant main effects [Distribution: *F*(1,87) = 1.132, *p* = 0.290; Modality; *F*(2,87) = 1.634, *p* = 0.201; Test Block *F*(1,87) = 1.345, *p* = 0.249]. Interactions between Modality and Distribution also proved insignificant [*F*(2,87) = 0.538, *p* = 0.586; three-way interaction with Block, *F*(2,87) = 0.792, *p* = 0.456].

Because other studies using looking time paradigms with infants often find an effect of learning only in one testing block (e.g., Feldman et al., 2013; Yeung and Nazzi, 2014) we went on to explore our findings by assessing difference scores in the first block. Again, we did not observe any effect of training on difference scores [Modality, *F*(2,87) = 1.171, *p* = 0.315; Distribution, *F*(1,87) = 0.609, *p* = 0.437; interaction between Modality and Distribution, *F*(2,87) = 0.214, *p* = 0.808].

Recall that according to the distributional learning hypothesis (e.g., Maye et al., 2002), we expect greater difference scores after two-peaked training than after one-peaked learning. According to the intersensory redundancy hypothesis (e.g., Bahrick and Lickliter, 2012), infants who saw and heard the vowel continuum had most evidence to learn the contrast. To explore whether any of the groups were successful in learning the contrast, we calculated *t*-tests on difference scores against the chance value of zero for each modality condition and distribution condition separately (**Table 2**). The criterion for finding significant discrimination then changes to a *p*-value of 1–0.95^1/6^ = 0.0085. Robust discrimination of the vowel contrast was found only for the infants in the two-peaked, multimodal training group [*t*(17) = 2.979, *p* = 0.0084]. There is no evidence for robust discrimination of the vowel contrast in any of the other five groups (all *p*’s > 0.334). Note that for a credible effect of training modality and distribution on discrimination, a group difference would have been required (e.g., better discrimination for infants in one group than for the other groups).

**Table 2:** Difference scores and their significance against zero for each condition.

Modality	Distribution	Mean difference (ms)	SE of mean (ms)	df	*t*	*p*
Multimodal	1-peaked	580	634	17	0.915	0.373
	2-peaked	1291	433	17	2.979	0.008
Visual	1-peaked	-26	667	13	-0.039	0.969
	2-peaked	-109	758	14	-0.144	0.888
Auditory	1-peaked	55	832	14	0.066	0.948
	2-peaked	720	715	12	1.006	0.334

### Infants’ Visual Scanning of the Face during Training

**FIGURE 3:**
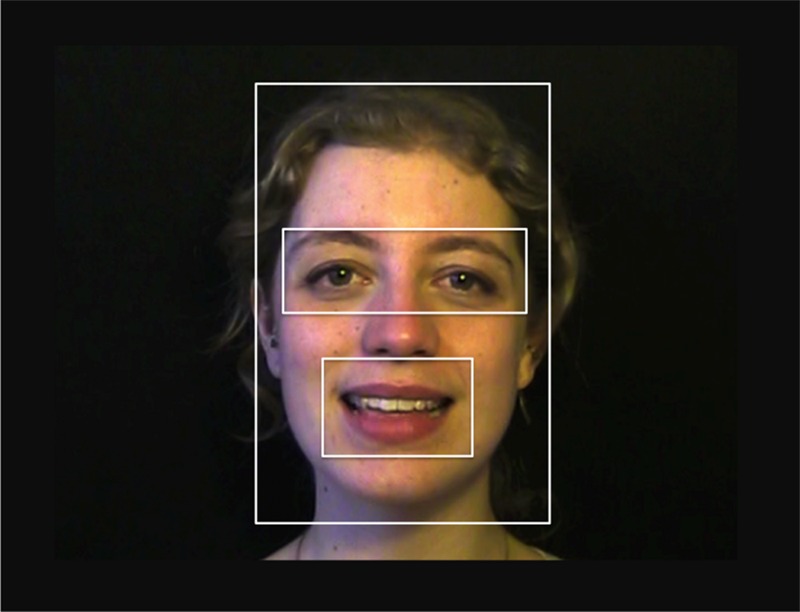
**The four regions of interest: eyes, mouth, rest of the face, rest of the screen**.

To investigate infants’ looking behavior over the course of training, we assigned locations of each eye gaze to one ROI as shown in **Figure 3**: the mouth area, the eyes, the rest of the face, and the rest of the screen. For each training block separately, we then calculated the proportion of looking time spent in the mouth and eyes areas relative to total face area. For each ROI, we performed a repeated-measures analysis of variance on these proportions across training, with training block (1 or 2) as a within-subjects factor and Modality (only multimodal and visual) and Distribution (one- or two-peaked) as between-subjects factors. One infant from the two-peaked visual training group had to be excluded from the analyses because this child did not fixate the face during the second block of training.

**FIGURE 4:**
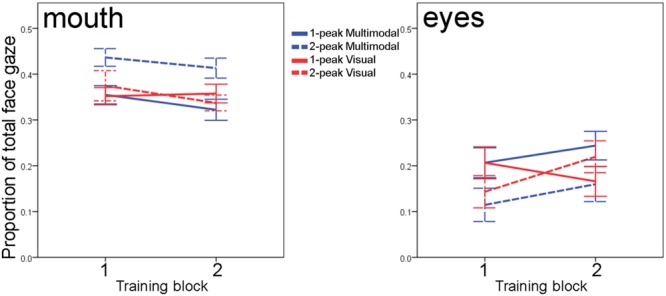
**Infants’ fixations to the speaker’s mouth **(left)** and eyes **(right)** calculated as proportions to total face gaze, for each of the two blocks of the training phase.** Solid lines vs. dashed lines reflect 1-peaked vs. 2-peaked trainings; color differentiates between multimodal (blue) and visual (red) trainings. Error bars denote one standard error of the mean.

For the mouth region, there was a marginal effect of training block [*F*(1,60) = 3.61, *p* = 0.062], which did not interact with any between-subject factors [all *F*(1,60) < 1.4; all *p* > 0.25]: overall, infants slightly decreased their looks to the mouth area in the second block. Irrespective of the course of the training, however, we observed a main effect of Distribution [*F*(1,60) = 5.29, *p* = 0.025] and an interaction between Modality and Distribution [*F*(1,60) = 5.01; *p* = 0.029]; the main effect of Modality was insignificant [*F*(1,60) = 1.92; *p* = 0.171]. The main effect of Distribution indicated that infants in the two-peaked conditions fixated the mouth area more often than infants in the one-peaked conditions, but this main effect was mainly driven by looking performance in the two-peaked multimodal group, as **Figure 4** shows: across training, it was the two-peaked multimodal condition in which infants looked 7 to 9 percent more to the mouth than in the other three conditions. *Post hoc* analyses (with α set to 0.0167^3^) show that throughout training, the two-peaked multimodal group scanned the mouth more often than the one-peaked multimodal (mean difference 8.6%, 98.333% CI = 2.4 ∼ 14.8%, *p* = 0.0011) and more than the one-peaked or two-peaked visual groups (mean difference 7.0%, 98.333% CI = 0.4 ∼ 13.6%, *p* = 0.012; mean difference 6.9%, 98.333% CI = 0.3 ∼ 13.5%, *p* = 0.013, respectively). Thus, although we do not see the expected main effect of Modality, there is a significant interaction between Modality and Distribution on infants’ mouth fixations during training. This interaction highlights that Modality can affect infants’ looking behavior to the mouth, albeit in an indirect fashion, that is, it is dependent on the type of distribution infants received.

We also examined infants’ scanning of the eye area over the course of training. Although the infants increased their looks to the eye region, the main effect of training block on eye looks [*F*(1,60) = 3.10, *p* = 0.08] was not significant, and neither were the interactions of training block with Distribution and Modality or their interaction [all three *F*(1,60) < 3.46, all *p* > 0.06]. We also did not observe any main or interaction effects of Distribution and Modality [all *F*(1,60) < 2.26, *p* > 0.13]. Thus, in contrast to our analysis for the mouth area, it appears that infants’ fixations to the speaker’s eyes are not dependent on the type of training they received.

Together, our exploratory ROI-analyses show that independent of the type of training infants received, they show similar development over the course of the training: insignificant increases to the speaker’s eyes coupled with insignificant decreases to the speaker’s mouth. The type of training, however, affected how much infants were fixating the speaker’s mouth: throughout the training phase, infants in the two-peaked multimodal group continued to be more attentive to the speaker’s mouth movements than the other three groups. We did not observe such effects of training type when we focused on infants’ fixations of the speaker’s eyes.

## Discussion

In this paper, we set out to study the added value of visual articulations on infants’ learning of a novel vowel contrast. This contrast was presented in six different learning contexts: through multimodal or unimodal information, in either a one-peaked or two-peaked frequency distribution. On the basis of the intersensory redundancy hypothesis (e.g., Bahrick and Lickliter, 2012), we expected that infants in the two-peaked multimodal group would discriminate the vowel contrast better at test than infants in any other group. Further, we expected that group differences at test could be traced back to group differences during training, in particular their scanning behavior. Detailed ROI-analyses suggested that increased attention to the mouth (but not to the eyes) differentiated groups: only infants in the two-peaked multimodal group fixated the mouth area more than infants in other visual and multimodal conditions.

Under what circumstances can infants acquire a difficult phoneme contrast more easily – and when is it more difficult? As several other studies in the literature, our study finds no overall effect of two-peaked versus one-peaked statistical distributions on infants’ speech perception, although other studies *have* shown such effects (see the Introduction). We know that the absence of a looking time difference does not automatically imply a failure to discriminate (see Aslin, 2007). Such null results therefore yield no information if we want to establish the circumstances that render learning difficult. Positive evidence of infants’ discrimination ability, on the other hand, *can* be used to answer the question when phoneme learning is easier. There is now accumulating evidence, for instance, that besides auditory distributional information, additional congruent visual information improves learning of a phoneme contrast. For instance, infants’ sensitivity to a non-native vowel contrast can be improved with a short training phase that paired these vowels consistently with two distinct visual objects, although this only held for infants who went on to have larger vocabularies at 18 months (Ter Schure et al., in press). Also, observing simultaneously visual articulations affects discriminability of a (native) consonant contrast (Teinonen et al., 2008), and the congruence or incongruence between non-native sounds and visual articulations can even alter infants’ listening preferences (Danielson et al., 2015). Although in our study we did not observe an interaction effect between modality condition and distribution, we find that infants discriminate the vowel contrast after training with two-peaked visual plus auditory distributions. Our finding suggests that even after perceptual reorganization, infants are able to show sensitivity to a novel vowel contrast (at least under some conditions).

Since phoneme categories appear to be multimodally specified in the infant brain (e.g., Bristow et al., 2009), we expected that multimodal speech would enhance learning of a novel phonological contrast as compared to unimodal speech, as long as the distribution of speech sounds would indicate the existence of a novel contrast. In addition, we expected that infants would look longer at the mouth of the speaker during two-peaked training than during one-peaked training. Other research has shown that more looking to the mouth is linked to learning of a non-native contrast (Lewkowicz and Hansen-Tift, 2012; Tomalski et al., 2013; Pons et al., 2015). Lewkowicz and Hansen-Tift (2012) propose a developmental shift in infants’ scanning patterns when presented with audiovisual speech over the course of the 1st year. While infants at 4 and 6 months of age fixate the eyes more than the mouth, they attend more to the mouth of a speaker by 8 months, while 12-month-olds focus more on the eyes again. This developmental shift is only apparent when infants were tested with native speech; for non-native speech, infants keep looking more at the mouth, even at 12 months of age. Our analysis of gaze locations during training replicates these findings. The two-peaked multimodal group fixated on the speaker’s mouth more than the other groups. The difference between multimodal and visual groups shows that it was not just the synchrony between speech and sound that induced infants to look more at the articulations; infants in the visual groups also heard speech that was synchronous with the articulations, but the formant frequencies that were essential for vowel perception were removed. Therefore, the current findings support the idea that 8-month-old infants’ attention is captured by specific correlations between speech sounds and articulations and not by simple on- and offset synchrony. Further, the interaction between modality and distribution shows that increased attention to the mouth is contingent on the perceived familiarity with the speech signal; for infants in the one-peaked training condition, sounds and articulations were consistent with their native input, while for infants in the two-peaked training condition, the audiovisual distributions signaled an unfamiliar contrast that was inconsistent with their native input.

These findings on gaze location are in line with the intersensory redundancy hypothesis (Bahrick and Lickliter, 2012), which suggests that when overlapping cues (e.g., the articulations and vowels in this study) are available across senses, infants appear to focus on the shared information (that is, amodal properties). This in turn helps them to detect changes in these amodal properties. For example, infants detect changes in the rhythm of a tapping hammer more easily when they both hear and see the hammer tapping than when the rhythm is conveyed by only one of the modalities (Bahrick and Lickliter, 2000). Similarly, infants recognize emotional affect better when it is expressed by both face and voice than when it is expressed by just the face or just the voice (for a review, see Grossmann, 2010). Thus, redundant information across the senses can guide infants’ attention to relevant information.

In short, infants’ visual scanning during speech perception by 8 months appears to be mediated by the distribution of the *speech* input, and this reflects the multimodal nature of infants’ representations. Although non-blind hearing infants attend to both visual and auditory information when presented with multimodal speech, they appear to focus especially on visual information when the auditory information is unfamiliar. While our study found no overall effect of distributional learning or modality on infants’ phonetic discrimination, differences in infants’ scanning patterns reveal an intricate interplay between statistical distributions and visual and auditory information during phonetic learning.

## Conclusion

This study looked at the effects of statistical distributions and audiovisual information on infants’ attention and learning of a non-native vowel contrast by 8 months. Although we could not reliably establish that discrimination was influenced by the number of distributional peaks (one vs. two) or by the modality of stimulus presentation (auditory vs. visual vs. multimodal), the group that objectively received the best information about the contrast, namely the two-peaked multimodal group, successfully discriminated the two vowels, which indicates that learning of a non-native vowel contrast can occur by 8 months. Infants in the two-peaked multimodal group also looked significantly longer at the mouth of the speaking face than any of the other groups, which suggests that the overlapping information in face and voice can affect infants’ perception of speech.

## Author Contributions

SS: conception and design of the work, acquisition, analysis and interpretation of data, drafting the paper. CJ: major contribution to the interpretation of the data, analysis of more specific gaze location data together with the first author, co-authoring the manuscript text and adding to the intellectual content. PB: major contribution to conception and design of the work and the interpretation of the data, co-authoring the manuscript text and adding to the intellectual content.

## Conflict of Interest Statement

The authors declare that the research was conducted in the absence of any commercial or financial relationships that could be construed as a potential conflict of interest.

## Footnotes

1 Equivalent Rectangular Bandwidth, a psychoacoustic measure of frequency. Distances along the ERB scale roughly reflect perceived auditory differences.

2 We reasoned that our difference in visual display (e.g., seeing a moving mouth compared to a still hand) would a priori induce different scanning patterns, simply because a still hand is less captivating than a moving mouth, regardless of the type of auditory stimulus. Indeed, across training, infants from the auditory conditions look consistently around 8–11% less at the ‘mouth’ (hand) area than infants from the other two modality-conditions [repeated-measures ANOVA: main effect of Modality *F*(2,86) = 15.83, *p* < 0.001; main effect of distribution *F*(1.86) = 5.02, *p* = 0.028; interaction between Modality and Distribution *F*(2,86) = 2.65, *p* = 0.076, plus a main effect of training block *F*(1,86) = 12.38, *p* = 0.001; but no interactions with training block]. At the same time, infants from the auditory conditions look on average 9–10% more at the eyes area than infants from the other two modality conditions [Repeated measures ANOVA: main effect of modality *F*(2,86) = 6.46, *p* = 0.002, and main effect of training block *F*(1,86) = 8.21, *p* = 0.005; no other interactions]. The main effects of test block indicate that in the second block of the training phase infants decreased their looks at the mouth area while increased their looks at the eyes area. The main effect of distribution for the mouth-ROI illustrates that infants in the two-peaked conditions fixate the speaker’s ‘mouth’ on average 3% more than infants in the one-peaked conditions.

3 To keep the family wise Type-I error rate at 0.05 or below, a multiple-comparison correction factor of 3 (i.e., a per-comparison α of 0.05/3) suffices, although there are six *post hoc* comparisons among the four groups. This is because (1) in case all four true means are equal, the omnibus ANOVA over the four groups (*p* = 0.006) limits the family wise error rate to 0.05; (2) in case three of the true means are equal and the fourth differs from them, there are only three comparisons that could potentially yield a type-I error; (3) in case two of the true means are equal and differ from the other two, which are equal to each other, there are only two comparisons that could yield a type-I error; and (4) in case two of the true means are equal and the other two differ from them and from each other, there is only one comparison that could yield a type-I error. In all cases, the probability of finding one or more *p*-values below 0.05 for groups whose true means are equal, stays at or below 0.05 if the correction factor for multiple comparisons is 3. This reasoning for four groups is a generalization from Fisher’s explanation for his three-group Least Significant Difference method; for *N* groups, the correction factor, if the omnibus ANOVA test is significant, is (*N*-1)(*N*-2)/2, which is 1 for *N* = 3, and 3 for *N* = 4.
